# Genetic Polymorphisms as Risk Stratification Tool in Primary Preventive ICD Therapy

**DOI:** 10.5402/2011/457247

**Published:** 2011-04-18

**Authors:** Roman Laszlo, Mathias C. Busch, Jüergen Schreieck

**Affiliations:** ^1^Abteilung für Kardiologie und Kreislauferkrankungen, Klinikum der Eberhard-Karls-Universität Tübingen, 72076 Tübingen, Germany; ^2^Klinik und Poliklinik für Innere Medizin B, Klinikum der Ernst-Moritz-Arndt-Universität Greifswald 17475 Greifswald, Germany

## Abstract

More and more implantable cardioverter-defibrillators (ICDs) are implanted as primary prevention of sudden cardiac death (SCD). However, major problem in practice is to identify high-risk patients for SCD. Different methods for noninvasive risk stratification do not have a sufficient positive or negative predictive value. Since current approaches lead to implantation of ICDs in a large number of patients who will never suffer an arrhythmic event and simultaneously patients still die of SCD who currently did not seem eligible for primary preventive ICD implantation, there is a need for additional tools for risk stratification. Epidemiological studies point to a hereditary risk of SCD. Different susceptibility of each person concerning arrhythmogenic events might be explained by genetic polymorphisms. By obtaining an individual “pattern” of polymorphisms of genes encoding for proteins which are important in arrhythmogenesis in one patient, risk stratification in primary prevention of SCD might by improved.

## 1. Introduction

Sudden cardiac death (SCD) is one of the most frequent causes of death [1]. Since 1980, implantable cardioverter-defibrillators (ICDs) are available as an effective, continuously improved therapy option [2]. Within the last thirty years, initially sole secondary preventive indications for implantation of an ICD have been expanded: more and more ICDs are implanted as primary prevention of SCD [3]. However, major problem in clinical practice is to identify high-risk patients for SCD. Since—on the one hand—evidence actually available [3] leads to implantation of ICDs in a large number of patients who will never suffer an arrhythmic event and—on the other hand—patients still die of SCD who did not seem eligible for ICD implantation, there is a profound need for additional tools for risk stratification.

## 2. Epidemiology of Sudden Cardiac Death

Epidemiologic studies report of more than 300000 SCDs/annum in the United States [1]. SCD is defined as unexpected death of cardiac cause within one hour after onset of any symptoms [4]. SCD occurs in newborn to 6-month old children (“sudden infant death syndrome”), and incidence of the disease peaks again in 45-to-75-year olds [1, 5]. 

In one third of all patients with coronary heart disease (CHD), SCD is the first clinical manifestation of the disease. 80% of all SCDs are caused by CHD, 20% by cardiomyopathies (dilated or hypertrophic) and, respectively, electrical diseases of the heart (long QT, short QT, Brugada-syndrome). 75% of patients with SCD are male [1]. 

Contrary to common assumptions, acute myocardial infarction can be found in only 20% of all patients with SCD. However, SCD is mostly caused by an electrical instability of ventricular myocardium which in turn is often generated by chronic ventricular ischemia [6]. 

Compared to normal population, risk of SCD is increased 2–4 times in patients with a high risk for CHD, 4–6 times in patients with apparent CHD and rises up to 6–9 if these patients develop congestive heart failure (CHF) [4].

## 3. Pathophysiology of Sudden Cardiac Death

Electrical instability of ventricular myocardium and consecutive arrhythmias leading to SCD is a result of complex interactions between triggering events and an arrhythmogenic substrate (often diseased myocardium) [7]. In most patients with SCD, ventricular fibrillation degenerated from precedent ventricular tachycardia can be found as terminal arrhythmia [8]. 

Lethal arrhythmia can be triggered by acute myocardial ischemia. Ventricular scar caused by previous myocardial infarction can either act as a trigger without manifest ischemia [7, 9] or as a substrate due to isolated regions of myocytes with slow conduction properties (e.g., endocardial cells which survived myocardial infarction due to nutrition and oxygenation from intracavitary blood) therefore predisposing re-entry circuits for sustained ventricular tachycardias [7].

Besides focal autonomy and triggered activity due to afterdepolarisations, re-entry is considered as the main mechanism for ventricular tachycardia in patients with CHD [10, 11]. 

SCD due to bradycardia, asystole, or pulseless electrical activity can be found more often in patients with severe structural heart disease [12].

## 4. Therapy of Sudden Cardiac Death

In the 1980s, antiarrhythmics were used in patients (mainly after myocardial infarction) with high-risk for SCD in order to prevent malign tachycardias. However, these drugs showed a considerable, sometimes fatal proarrhythmic potential in several large randomized studies [13]. Antiarrhythmics including amiodarone which is regarded as most effective and accordingly safe [14–16] are also inferior compared to today's standard treatment of SCD, the implantable cardioverter/defibrillator (ICD [2]). Thus, sole (i.e., without concomitant implantation of an ICD) primary preventive antiarrhythmic therapy in order to anticipate SCD is obsolete [3]. 

On the one hand, incidence of malign tachycardias within an observation period is also high in patients currently considered as high-risk suffering SCD, but—on the other hand—these patients represent only a small percentage of all cases of SCD. In the majority of patients with SCD, only little or even no clinical signs for a potential occurrence of lethal arrhythmias can be observed [17].

Besides reliable and accordingly feasible methods to identify high risk patients for SCD, this discrepancy depicts the main problem of primary prevention of SCD with ICDs.

## 5. Current Problems in Primary Preventive ICD Therapy

Current primary prevention of SCD (implantation of an ICD) is mostly limited to patients with considered high-risk for SCD. According to present level of knowledge, this high risk collective is mainly defined by having reduced left ventricular ejection fraction (LV-EF) and/or clinical symptoms of CHF. Primary preventive implantation of an ICD can be considered in all patients with severely reduced LV-EF (<35%) and little or moderate symptoms (NYHA II-III) or even independent of symptomatology for patients with ischemic cardiomyopathy [3].

However, problems are implicated by using reduced LV-EF to identify candidates for ICD implantation: first, after myocardial infarction, most cases of SCD can be found in patients with better preserved LV-EF [18–20]. Second, only a minority of patients received an ICD due to severely reduced LV-EF de facto suffer from life-threatening arrhythmias [16, 21, 22]. Finally, at as far as 50% of all patients with CHF, cause of death is progressive heart failure but not malign ventricular arrhythmias [23]. 

As well as antiarrhythmics, ICDs are also not free of risks: proportion of infectious complications, inadequate shock deliveries, and other device malfunctions is considerable [24–26]. Despite proven efficiency of primary prevention of SCD [14, 16, 21], ICD implantation is still expensive and depicts a significant burden of health care system [27].

A multiplicity of invasive and, respectively, non-invasive methods in order to better select patients for primary preventive ICD implantation was developed. Some approaches are based on signal-averaged ECGs [28], QT variability, heart rate variability, T-wave variability, microvolt T-wave alternans, heart rate turbulence, deceleration capacity, or the so-called Wedensky modulation index [29]. Holter-ECG can be also used to evaluate the incidence of nonsustained ventricular tachycardias [30] as well as invasive electrophysiological analyses (programmed ventricular stimulation [30]). Ventricular arrhythmogenic substrate can be described using cardiac magnetic resonance imaging [31]. Finally, some laboratory parameters like BNP [32] or CRP [33] seem to predict the risk for SCD in some patients. 

All these approaches—neither alone nor combined [19]—do not have an adequate positive or rather negative predictive value. Thus, LV-EF and symptoms of CHF (NYHA classification) are still mainly used to legitimate primary preventive ICD implantation with the most additional denotations of the above-mentioned methods [3, 34].

In summary, delineated problems of current primary preventive ICD therapy underline the need for new methods for a better risk stratification of primary prevention of SCD.

## 6. Genetic Predisposition for Sudden Cardiac Death

In comparison with normal population, first-degree relatives of patients with SCD have an increased risk for this purpose (relative risk 1.5–2.7) [35–37].

Family history concerning SCD is more often positive in patients with ventricular fibrillation up to the time of first myocardial infarction (odds ratio 2.7) [38]. 

Positive family history potentiates the individual risk in patients suffering from certain diseases predisposing for SCD, as for example, fivefold in patient with hypertrophic cardiomyopathy [39].

Taken together, these data speak for a familiar accumulation of SCD, maybe because of common genes influencing individual vulnerability to life-threatening arrhythmias independent of a familiar predisposition for ischemic heart disease in general.

In addition, dependence of risk on the number of affected family members (the more, the higher) is compatible with a complex genetic architecture where predisposing alleles increase the risk for SCD in case of arrhythmogenic environment variables in an additive or synergistic way [40, 41].

## 7. Genetic Polymorphisms as a Risk Stratification Tool in Primary Preventive ICD Therapy

Genetic variations (so called polymorphisms) are the basis of human uniqueness. Specific pros and cons concerning state of health or susceptibility to certain diseases would not be expected from variations of genes coding, for example, for the physiognomy of a single person. However, occurrence of variations of genes coding for proteins essentially for physiological functions (e.g., cellular calcium homoeostasis or function of ion channels) might be disadvantageous in terms of, for example, proarrhythmic compensatory mechanisms during myocardial ischemia, disbalance of electrolytes, or also after drug intake. Therefore, polymorphisms of these genes might explain different susceptibility of persons concerning arrhythmogenic events and may suit for non-invasive risk stratification of SCD: if, for example, two patients are considered to have the same risk for SCD using currently available approaches for risk stratification but then in only one patient SCD actually occurs, this differential outcome might be explained by genetic polymorphisms. 

As a clinical aim, identification of several polymorphisms in a patient which might play a role in arrhythmogenesis may result in an individual genetic “polymorphism pattern” in order to characterize the individual risk for occurrence of arrhythmic events. Besides the well-established parameters (LV-EF, NYHA class), this “genetic pattern” might then act as another helpful tool in decision-making concerning primary preventive ICD implantation.

Associations of many polymorphisms—most notably of genes coding for ion channels—and SCD were already reported [42].

Besides cardiac ion channels, molecular signaling cascades like, for example, the beta-1-receptor cascade and accordingly the renin-angiotensin-aldosterone-system (RAAS) play an important role concerning regulation of cardiac electrophysiology. 

On the one hand, many polymorphisms of these signaling cascades with unclear function importance are known; on the other hand there are others with relevance demonstrated in vitro and others associated with the incidence of arrhythmias in patients with CHF. 

For example, sensitivity of the beta-1-receptor in vitro [43] and heart rate [44] and survival [45] in a small cohort of patients with CHF is influenced by the Ser49Gly polymorphism (frequency of occurrence in normal population 22%). In transfected fibroblasts, the Arg389Gly polymorphism (frequency of occurrence in normal population 30%) causes a threefold increase of the activity of the adenylate cyclase [46] and seems to enhance the incidence of arrhythmias in patients with CHF [47].

Development of an arrhythmic cardiac fibrosis is essentially influenced by the RAAS [48]. It has been shown that patients with CHF rather die of a progressive heart failure than of a malign arrhythmia if a common polymorphism of angiotensin can be detected [49]. Further RAAS polymorphisms were identified but not yet validated in patients with CHF. 

Intracellular calcium overload is a well-known cause of triggered ventricular arrhythmias as a result of delayed afterdepolarisations [10]. Polymorphisms of proteins important for regulation of intracellular calcium homoeostasis were also described (e.g., Glu692Val (frequency of occurrence in normal population 5%) of the Na+/Ca2+ exchanger, Gln2958Arg (frequency of occurrence in normal population 20–30%) of the cardiac ryanodine type 2 receptor). As cellular calcium homoeostasis is per se defective in patients with CHF, it can be speculated that these polymorphisms cause an increased risk for occurrence of malign tachycardias.

## 8. Conclusion

As a summary, utilization of the genetic information for non-invasive risk stratification of SCD seems to be feasible. By obtaining an individual “pattern” of polymorphisms of genes encoding for proteins which are important in arrhythmogenesis in one patient, risk stratification in primary prevention of SCD might by improved, and current problems in primary preventive ICD therapy might be minimized.
